# Patient Satisfaction with Community Health Service Centers as Gatekeepers and the Influencing Factors: A Cross-Sectional Study in Shenzhen, China

**DOI:** 10.1371/journal.pone.0161683

**Published:** 2016-08-23

**Authors:** Jiang Wu, Shengchao Zhang, Huiqing Chen, Yingyu Lin, Xiaoxin Dong, Xiaoxu Yin, Zuxun Lu, Shiyi Cao

**Affiliations:** 1 Baoan Central Hospital of Shenzhen, Shenzhen, China; 2 School of Public Health, Tongji Medical College, Huazhong University of Science and Technology, Wuhan, China; Tilburg University, NETHERLANDS

## Abstract

**Purpose:**

Shenzhen is the first pilot city in China implementing the gatekeeper policy, with community health service (CHS) centers as the gatekeepers. We aim to investigate patient satisfaction with this policy and its influencing factors in Shenzhen.

**Methods:**

3,848 patients visiting eight CHS centers in Shenzhen of China between May 1 and July 28, 2013 were recruited. We interviewed them using a structured questionnaire to investigate their satisfaction with the gatekeeper policy of CHS. Multivariable logistic regression models were used to identify influencing factors.

**Results:**

Of the respondents, 28.17%, 47.27% and 24.56% were satisfied with, neutral to, and not satisfied with the gatekeeper policy respectively. Patient satisfaction with this policy was found to be associated with education level, familiarity with the policy, referral experience, satisfaction with convenience of seeing a doctor, satisfaction with waiting time, satisfaction with medical facility, satisfaction with general medical practitioners’ professional skill, and proportion of expense reimbursed.

**Conclusions:**

Our investigation shows that patient satisfaction with the gatekeeper policy was low. To improve patient satisfaction, efforts should be made to increase the convenience of seeing a doctor in community, shorten waiting time, improve general medical practitioners’ professional skill, and increase proportion of expense reimbursement.

## Introduction

Community health service (CHS) is an important means to provide a comprehensive, universal, equitable, and affordable healthcare service worldwide [1, 2]. To ensure full play to the functions of CHS, many developed countries adopt the gatekeeper policy, with CHS providers as gatekeepers to attract, guide, or channel residents to seek healthcare in CHS institutions [3–6].

In China, after several decades’ health care reforms, the government has now taken a step forward in strengthening the tiered medical system where CHS institutions care is for common illnesses and hospitals care is for serious diseases, of which the gatekeeper function of CHS is a key and contentious point. The gatekeeper policy of CHS requires a patient to visit a healthcare provider in his designated CHS institution first, and, if necessary, get the provider’s referral before seeing a specialist or going to a hospital. This policy is widely implemented in many developed countries, such as United Kingdom, Spain, Switzerland, and the Netherlands [7–10], and has been proved that it can improve healthcare continuity and coordination, reduce inappropriate use of specialty care and hospitalization, and reduce overall health expenditure [11–13].

However, even though China has invested in building the network of CHS for nearly two decades [14], CHS falls far short in meeting the expectation as the nation’s primary care providers. Chinese patients have the freedom to choose any medical institutions, including CHS institutions and all kinds of hospitals, as their first recourse for treatment [2]. Many patients, even with common diseases, are more inclined to see doctors in hospitals, aggravating the difficulty and expensiveness of getting medical treatment and going against the fundamental principle of the tiered medical system. For these reasons, China started experimenting the gatekeeper policy.

To evaluate the feasibility of this policy in China, Shenzhen, a developed south city neighboring Hong Kong, was selected as the first pilot city to implement the gatekeeper policy in 2006. In Shenzhen, migrant workers coming from all over the country account for over 80% of the city’s current population. Since 2006, a labor health insurance system has offered coverage to all migrant workers and their families, and each enrollee is bonded to a CHS center for first health service and is required to get his or her designated CHS center for referral for care elsewhere. Enrollees who seek care elsewhere without referrals of CHS institutions are to pay for themselves out-of-pocket. There are many possible reasons that patients are dissatisfied with this policy, first of all, because of restrictions on their free choices.

Taken into consideration of the importance of patient satisfaction on implementing and spreading this gatekeeper policy in China, we conducted this study to assess patient satisfaction with this policy and its influencing factors in Shenzhen, in a population that has had eight years of experience with this policy.

## Methods

### Ethics statement

The study protocol was approved by the Research Ethics Committee in Tongji Medical College, Huazhong University of Science and Technology, Wuhan, China. All the participants read the purpose statement of the investigation and provided written informed consents. We conducted the present investigation in accordance with the approved protocol.

### Participants and sampling

Our study design was cross-sectional. Data were collected from May 1 to July 28 2013 in the city of Shenzhen, Guangdong Province (Southern China). Multistage sampling was conducted to recruit participants. We selected Baoan District, the largest administrative district of Shenzhen, as our study site, and four sub-districts in Baoan District were randomly chosen. In each sub-district, we randomly selected two CHS centers. In each CHS center, we interviewed 500 outpatients who were from a convenience sample of patients at the CHS at the time of the visit. Outpatients younger than 18 years old were excluded. A total of 4000 participants signed informed consent and filled in the questionnaires. Of the 4000 questionnaires, 3848 were completed and collected, and 152 questionnaires were excluded because of too many missing data. The overall response rate was 96.20%.

### Questionnaire design

There were no standard questionnaires for our study. Therefore, we designed the questionnaire ourselves in view of our study purpose. Our structured questionnaire contained four sections: socio-demographic information, health status and health-seeking behavior, awareness and understanding of the gatekeeper policy, and satisfaction with CHS and the gatekeeper policy.

Socio-demographic information included gender, age, marital status, education level, and income per month. Health status and health-seeking behavior comprised of self-reported health status, chronic non-communicable diseases, and referral experience. Items about awareness and understanding of the gatekeeper policy of CHS contained awareness of the gatekeeper policy, familiarity with relevant polity about the gatekeeper policy, and view of CHS institutions’ condition and capacity of implementing the gatekeeper policy. Items of satisfaction with CHS center and the gatekeeper policy covered satisfaction with convenience of seeing a doctor, waiting time, environment, medical facility, technical level and service attitude of general practitioners, proportion of expense reimbursement, difference of medical expenses between CHS center and general hospital, and satisfaction with the gatekeeper policy. All satisfaction measures were scored using a single 5-point Likert scale [15] ranging from ‘‘very satisfied” to ‘‘very unsatisfied” and coded in values from 1 to 5.

### Investigation process, data collection, and quality control

The study was organized and coordinated by Huazhong University of Science and Technology and Health Bureau of Baoan District. Following the study protocol, senior investigators from Huazhong University of Science and Technology provided training to junior investigators, and, the junior investigators carried out the survey on the patients at the exit of CHS centers. The senior investigators checked the collected questionnaires daily to conduct quality control. The data was double-blindly entered into the database by two different researchers using EpiData 3.0 to guarantee the correctness.

### Statistical analyses

All statistical analyses were performed using the Statistical Package for Social Sciences Version 13.0 for Windows. Descriptive analysis was carried out for socio-demographics data, self-reported physical health status, care seeking behavior, and satisfaction with CHS. Patient satisfaction with the gatekeeper policy was calculated and compared by socio-demographic characteristics, health status, care seeking behavior, and satisfaction with CHS. Chi-square tests were conducted to compare the satisfaction with the gatekeeper policy between subgroups. Multivariable logistic regression analysis (the entry method of independence variables is ‘Enter’) was used to analyze the influencing factors of patient satisfaction with the gatekeeper policy, with socio-demographic characteristics, health status, care seeking behavior, and satisfaction with CHS as the independent variables. We included only the variables as the independent variables if the results of corresponding bivariate analysis were statistically significant. Adjusted odds ratios (ORs) and *P* values were calculated. For all comparisons, differences were tested using two-tailed tests and *p* values less than 0.05 were considered statistically significant.

## Results

### Demographic characteristics of the participants

Most of the participants were younger than 40 years old (85.88%) and only 0.18% respondents were 66 years old or above. More than half of the participants were females (56.51%). Of the participants, 73.23%, 25.94%, and 0.78% were married, unmarried, and divorced respectively. The educational level of most respondents was middle school (83.10%). More than 90% of the participants had incomes per month less than 4,917 RMB (Table 1).

**Table 1:** Socio-demographic information of the participants and association with satisfaction with the gatekeeper policy.

Variables		%	Unsatisfied or neutral		Satisfied		Χ^2^	*P*
			N	%	N	%		
**Gender**	**Male**	43.49%	1160	69.71	504	30.29	6.66	0.0099
	**Female**	56.51%	1589	73.50	573	26.50		
**Age**	**18~30**	54.03%	1511	73.00	559	27.00	8.40	0.0384
	**31~40**	31.85%	877	71.89	343	28.11		
	**41~65**	13.94%	357	66.85	177	33.15		
	**66 and above**	0.18%	4	57.14	3	42.86		
**Marital status**	**Unmarried**	25.94%	701	72.34	268	27.66	2.26	0.5195
	**Married**	73.23%	1961	71.70	774	28.30		
	**Divorced**	0.75%	21	75.00	7	25.00		
	**Others**	0.08%	3	100.00	0	0.00		
**Educational level**	**Primary school or below**	7.11%	177	65.80	92	34.20	10.05	0.0182
	**Middle school**	83.10%	2266	72.10	877	27.90		
	**Junior college**	7.88%	216	72.48	82	27.52		
	**Regular college or above**	1.90%	60	83.33	12	16.67		
**Income per month**	**2458 or below**	43.40%	1191	71.32	479	28.68	0.54	0.9107
	**2459~4917**	52.65%	1463	72.21	563	27.79		
	**4917~7376**	3.27%	92	73.02	34	26.98		
	**7376 and above**	0.68%	18	69.23	8	30.77		

Abbreviations: CHS = community health service

### Patients’ satisfaction with the gatekeeper policy and its influencing factors

Of the respondents, 28.17%, 47.27% and 24.56% were satisfied with, neutral to, and not satisfied with the gatekeeper policy respectively. Bivariate analysis shows that gender, age, and educational level were significantly associated with satisfaction with the gatekeeper policy (Table 1). Whether the participants had chronic non-communicable disease, whether they had heard of the policy, whether they were familiar with relevant rules about the policy, and referral experience were also associated with the satisfaction. In addition, the satisfaction with CHS (including convenience of seeing a doctor, waiting time, medical environment, medical facility, general medical practitioners’ professional skill, service attitude, and proportion of expense reimbursement) had a statistically significant association with the satisfaction with the policy. (Table 2)

**Table 2:** Health status and health-seeking behavior, awareness and understanding of the gatekeeper policy, and satisfaction with CHS.

Variables		Unsatisfied or neutral		Satisfied		Χ^2^	*P*
		N	%	N	%		
Self-reported health status	Good	1153	73.11	424	26.89	2.47	0.2902
	Neutral	1426	71.73	562	28.27		
	Bad	156	68.42	72	31.58		
Chronic non-communicable disease	No	2088	73.11	768	26.89	8.97	0.0027
	Yes	676	68.15	316	31.85		
Have you heard of The gatekeeper policy	Yes	772	62.46	464	37.54	95.61	< .0001
	No	1835	77.79	524	22.21		
Are you familiar with relevant rules of the gatekeeper policy	Familiar	64	40.00	96	60.00	133.60	< .0001
	Neutral	1237	68.23	576	31.77		
	Not familiar	1448	78.57	395	21.43		
Do you think that community health centers have the condition and capacity of implementing the gatekeeper policy	Yes	606	60.60	394	39.40	9.27	0.0097
	No	48	67.61	23	32.39		
	Don’t know	272	69.04	122	30.96		
Referral experience	Yes	747	68.72	340	31.28	5.63	0.0176
	No	1487	72.75	557	27.25		
Convenience of seeing a doctor	Unsatisfied or neutral	1162	82.35	249	17.65	120.53	< .0001
	Satisfied	1593	65.83	827	34.17		
Waiting time	Unsatisfied or neutral	2038	82.05	446	17.95	368.34	< .0001
	Satisfied	712	52.82	636	47.18		
Medical environment	Unsatisfied or neutral	1528	84.19	287	15.81	255.39	< .0001
	Satisfied	1227	60.95	786	39.05		
Medical facility	Unsatisfied or neutral	1922	84.45	354	15.55	443.35	< .0001
	Satisfied	818	53.19	720	46.81		
Medical skill level of CHS providers	Unsatisfied or neutral	1175	88.68	150	11.32	284.30	< .0001
	Satisfied	1574	62.91	928	37.09		
Service attitude	Unsatisfied or neutral	880	86.11	142	13.89	139.46	< .0001
	Satisfied	1876	66.71	936	33.29		
Proportion of expense reimbursement	Unsatisfied or neutral	1378	88.16	185	11.84	350.73	< .0001
	Satisfied	1369	60.44	896	39.56		
The difference in medical expenses between CHS center and general hospital	Unsatisfied or neutral	1444	89.25	174	10.75	421.23	< .0001
	Satisfied	1302	59.02	904	40.98		

Abbreviations: CHS = community health service

Table 3 demonstrates the adjusted ORs and *P* values for satisfaction with the gatekeeper policy. Compared with patients with the educational level of primary school or below, those with the educational level of middle school, junior college, and regular collage or above had higher odds for being satisfied with the gatekeeper policy. Patient satisfaction with this policy was associated with education level, familiarity with the policy, referral experience, convenience of seeing a doctor, waiting time, medical facility, general medical practitioners’ professional skill, and proportion of expense reimbursement.

**Table 3:** Logistic regression analysis on potential influence factors of being satisfied with the gatekeeper policy.

Variables	Partial regression coefficient (B)	*P* value	Odds Ratio	95% CI for Odds Ratio	
				Lower	Upper
**Gender (Ref. = Male)**					
Female	-0.124	0.417	0.884	0.656	1.191
**Age (Ref. = 18–30)**					
31~40	1.970	0.149	7.167	0.492	104.305
41~65	1.888	0.166	6.607	0.457	95.558
66 and above	1.814	0.183	6.137	0.424	88.808
**Educational level (Ref. = Primary school or below)**					
Middle school	3.273	0.021	10.925	1.134	110.567
Junior college	2.571	0.038	9.946	1.107	104.622
Regular college or above	2.446	0.046	9.213	1.059	97.342
**Chronic non-communicable disease (Ref. = Yes)**					
No	0.718	0.207	2.051	0.671	6.266
**Have you hear of the gatekeeper policy of CHS (Ref. = Yes)**					
No	-0.575	0.000	0.563	0.276	0.845
**Are you familiar with relevant policy of the gatekeeper policy of CHS (Ref. = No)**					
Neutral	0.590	0.001	1.804	1.624	2.227
Yes	0.731	0.000	1.942	1.728	2.293
**Do you think that community health centers have the condition and capacity of implementing the gatekeeper policy (Ref. = Yes)**					
No	0.574	0.351	1.776	0.845	3.571
**Referral experience (Ref. = Yes)**					
No	-0.765	0.000	0.361		
**Convenience of seeing a doctor (Ref. = Unsatisfied or neutral)**					
Satisfied	0.492	0.000	1.401	1.317	1.612
**Waiting time (Ref. = Unsatisfied or neutral)**					
Satisfied	0.221	0.000	1.247	1.113	1.396
**Medical environment (Ref. = Unsatisfied or neutral)**					
Satisfied	0.013	0.834	1.013	0.897	1.144
**Medical facility (Ref. = Unsatisfied or neutral)**					
Satisfied	0.272	0.000	1.313	1.164	1.482
**Medical level (Ref. = Unsatisfied or neutral)**					
Satisfied	0.266	0.000	1.305	1.139	1.495
**Service attitude (Ref. = Unsatisfied or neutral)**					
Satisfied	-0.066	0.358	0.936	0.814	1.077
**Proportion of expense reimbursement (Ref. = Unsatisfied or neutral)**					
Satisfied	0.277	0.000	1.319	1.172	1.486
**The difference of medical expenses between community health center and general hospital (Ref. = Unsatisfied or neutral)**					
Satisfied	0.526	0.000	1.693	1.506	1.903

Abbreviations: Ref. = Reference; CHS = Community health service; CI = Confidence interval

## Discussion

After China started the market-oriented economic reforms in the early 1980s, the widespread, three-tiered healthcare system that offered preliminary yet comprehensive, equitable health services to all collapsed quickly [16]. The disintegration of CHS network resulted in serious lack of access to basic care in general and lack of affordability when patients have to seek care in hospitals in cities [17]. For over a decade, China has invested a great amount of resources to rebuild the three-tier system, with the focus squarely on the bottom tier, the community health service centers. However, China, and some other developing countries, are now faced with the same, persistent problem in maintaining the tiered medical system and the CHS providers, and gatekeeper system with CHS being the designated gatekeepers is considered the most viable and effective solution to this problem. [18]. One obvious drawback of the gatekeeper policy is the restriction of patient choices, and to implement the policy widely and for a long run, policy makers must understand how to improve patient satisfaction given the core principles of any gatekeeper system. Our investigation on patient satisfaction with the gatekeeper policy implemented in Shenzhen, China, and exploration of the influencing factors, would provide important reference information to improve the implementation of the policy in China and other developing countries.

Overall, we found that only 28.17% of patients were satisfied with the gatekeeper policy. As is well-known, the low satisfaction represents the residents’ passive attitude to the gatekeeper policy of CHS, which can potentially affect the implementation effect of the policy. The emphasis of implementation and extension of the gatekeeper policy should be focusing on residents’ (especially patients) benefit. We also found that unfamiliarity with this gatekeeper policy, inconvenience of seeing a doctor in community, longer waiting time, lower medical skill level of CHS providers, and lower proportion of expense reimbursement in CHS institutions increase the odds of patient dissatisfaction with the gatekeeper policy. Our results suggest that the gatekeeper policy implemented in Shenzhen could face substantial resistance among patients and general population. Our analyses on the influencing factors point to several remedies that could improve patient satisfaction with the policy, including more targeted education about the policy, improving CHS services in terms of convenience, waiting time and better staffing. For the short term, increasing the reimbursement rate for services at CHS centers and/or decrease reimbursement for hospital care may improve patient satisfaction with the gatekeeper policy.

Up to now, this is the first original investigation on community residents’ satisfaction degree with the gatekeeper policy of CHS, although various policies similar to that of Shenzhen have been implemented in many other cities of China, such as Beijing, Zhuhai, and Nanjing [19–22]. It will play an important role in popularize the gatekeeper policy of CHS in China. However, some limitations in the present study should be noted. Firstly, the potential influence factors of patient satisfaction with the gatekeeper policy are possibly more than those we investigated, but we failed to identify all of them. Secondly, our sample was from one city and relatively small, although this city is one of the most developed cities in China with high proportion of floating population who are from various other cities in China. More studies are needed to broaden the sample selection and include more potential factors influencing patient satisfaction with the gatekeeper policy, especially those factors for which specific interventions can be devised to improve patient acceptance of the policy.

Another concern should be mentioned about our study. In fact, our results regarding discontent with gatekeeping partly illustrate why few local government in China would require local resident populations with permanent residence registration to accept gatekeeping—as officials state privately, such a requirement might endanger social stability and residents would be resentful. Only the less powerful migrants can be forced to accept such a norm, and clearly do resent it. Other places such as Hangzhou and Shanghai have taken a more tender approach of “bribing” voluntary gatekeeping arrangements by lowering co-payments and facilitation referrals, etc., rather than making CHS first-contact care mandatory.

In summary, patient satisfaction with the gatekeeper policy in China, where CHS centers being the gatekeepers, was low. Educating the Chinese population about the policy, increasing the convenience of seeing a doctor in community, shortening waiting time, improving medical skill level of CHS providers, and increasing reimbursement for CHS services are essential to improving patient satisfaction with this policy.

## Supporting Information

S1 Appendix The minimal data set underlying the findings in this study. (RAR)
